# The impact of right bundle branch block and SIQIII-type patterns in determining risk levels in acute pulmonary embolism

**DOI:** 10.12688/f1000research.131758.1

**Published:** 2023-05-24

**Authors:** Majed Hassine, Mohamed Yassine Kallala, Ahmed Jamel, Ines Bouanene, Nidhal Bouchahda, Marouen Mahjoub, Kais Memmi, Najeh Ben Halima, Habib Gamra

**Affiliations:** 1 Cardiology A Department, Cardiothrombosis Research Laboratory, Fattouma Bourguiba University Hospital, Universite de Monastir, Monastir, Monastir, 5000, Tunisia; 2 Cardiology Department of Kairouan, Universite de Sousse, Sousse, Sousse, 5030, Tunisia; 3 Department of Epidemiology and Preventive Medicine, Fattouma Bourguiba Hospital, Universite de Monastir, Monastir, Monastir, 5000, Tunisia

**Keywords:** Electrocardiogram, Pulmonary embolism, Prognosis, Mortality

## Abstract

**Background:** Electrocardiography (ECG) findings in acute pulmonary embolism (PE) are known to be related to various right ventricular (RV) alterations. These abnormalities are not included in risk stratification algorithms despite emerging evidence of their association with patient outcomes. We aimed to analyze the impact of right bundle branch block (RBBB) and/or SIQIII patterns as indicators for determining the level of risk in patients with PE.

**Methods:** We performed a retrospective cohort study including all patients with confirmed acute PE hospitalized from January 2008 to December 2019 in two tertiary care cardiology departments. The first ECG taken at admission was selected and the analysis focused on the presence of a complete or an incomplete RBBB and SIQIII-type patterns.

**Results:** A total of 255 patients were divided into two groups: Group I (47.8%, n=122) included patients with PE without RBBB nor SIQIII patterns, and Group II (52.2%, n=133) included patients with RBBB and/or SIQIII patterns. Patients in group II presented significantly more frequently with acute right heart symptoms (45.1% *vs.* 18%, p<0.001) and cardiogenic shock at admission (31.6 *vs.* 4.1%, p<0.001). Echocardiographic parameters indicating right heart injury also occurred more significantly in group II patients (p<0.001). By univariate analysis, patients in group II were found to be significantly associated with in-hospital mortality (22.6 *vs.* 6.1%, p=0.002) and major cardiovascular events (MACEs) during hospitalization (43.3 *vs.* 13.7%, p<0.001). Multivariate logistic regression analysis identified five independent factors predictive of MACEs: SIQIII and/or RBBB, renal failure, positive troponin levels, RV dysfunction and right heart failure symptoms during initial presentation. Kaplan-Meier survival analysis identified the inclusion in Group II and the presence of SIQIII pattern as predictors of overall mortality (p<0.001).

**Conclusions:** Our study suggests an important and independent prognostic value of RBBB and SIQIII patterns and their usefulness in determining the outcome of PE patients.

## Introduction

Pulmonary embolism (PE) is a common and potentially life-threatening medical condition. It is estimated that thrombo-embolism affects over 1,000,000 people in the United States each year and results in over 25,000 deaths annually. ^1^

Risk stratification is important in determining the likelihood of poor outcomes for patients with acute PE. It dictates the attitude towards patients with PE from early discharge to urgent care in intensive care units. Thus, recent risk stratification algorithms rely on various clinical, laboratory, and imaging factors to estimate the risk of complications and guide treatment decisions. ^2^ A major area of uncertainty in the risk stratification of acute PE is the potential role of electrocardiography (ECG) abnormalities in predicting the risk of complications as several ECG abnormalities have been identified in patients with acute PE, including right ventricular strain and S1Q3T3 type pattern. ^3^^–^^5^ These changes are primarily related to right ventricular (RV) overload and reflect right ventricular dysfunction (RVD), injury and enlargement in patients with acute PE. ^6^^–^^9^ Furthermore, ECG abnormalities associated with acute PE are more likely to be present in patients with a confirmed diagnosis of PE. ^2^^,^^10^ Despite their potential prognostic value, none of these ECG changes are included in the current guidelines for PE risk stratification due to the lack of specificity. ^2^^,^^11^ Encouraging evidence has emerged showing the relationship between the right bundle branch block (RBBB) and SIQIII patterns in acute PE events with poor outcomes. ^10^^,^^12^^,^^13^ In addition, more research is needed to clarify the potential role of ECG abnormalities in the risk stratification of acute PE. By addressing these gaps in the literature, we can improve our ability to manage patients with this common and potentially life-threatening condition. ^12^^,^^14^

### Aim

Our study aimed to examine the impact of RBBB and/or SIQIII-type patterns as indicators for determining the level of risk in patients with acute PE.

## Methods

### Ethical considerations

This study was a retrospective observational cohort study that did not involve human testing and did not challenge human rights. The ethical approval was obtained retrospectively as we did not initially believe that this study needed ethical approval. This issue has been a subject of debate inside our team and that belief has since been revised and updated. Data collection for our study began in January 2011 in two Tunisian tertiary care cardiology departments: Cardiology A Department, Fattouma Bourguiba University Hospital affiliated to the University of Monastir and the Cardiology Department of Kairouan affiliated to the University of Sousse. Our study was retrospectively approved by the Research Ethics Committee of the Faculty of Medicine of Monastir (an independent organization under the aegis of the Tunisian Ministry of Public Health) under the number IORG 0009738 N°114/OMB 0990-0279. We obtained this approval on the 16 ^th^ of March 2023.

The initial approval to start the study was obtained by consensus from both research teams under the supervision of both heads of the aforementioned departments at the University of Monastir and University of Sousse. The study was performed in compliance with the Declaration of Helsinki. We ensured that participants’ privacy and confidentiality were maintained, and the study results were reported in a way that protected the participants’ identities.

Oral informed consent was obtained from patients, whenever possible. This verbal consent was obtained during initial hospitalization. Each patient was informed that their anonymized data could serve for future research purposes. We did not conceptualize the content of this article at the time of information gathering and we could not obtain this consent in patients initially presenting with critical conditions and fatal outcomes. Verbal consent was approved retrospectively when we received ethical approval and deemed adequate by the ethics committee.

### Study design

We conducted a retrospective cohort study. We included all patients with confirmed acute PE hospitalized from January 10 ^th^, 2008, to December 31 ^st^, 2019, in two Tunisian tertiary care cardiology departments: Cardiology A Department, Fattouma Bourguiba University Hospital affiliated to the University of Monastir and the Cardiology Department of Kairouan affiliated to the University of Sousse. The data were gathered by reviewing patients’ hospital records from each hospitalization, supplemented with comprehensive in-hospital assessments and follow-up interviews conducted either in person or *via* telephone.

PE was diagnosed primarily by either computed tomography (CT) or scintigraphic ventilation-perfusion (V/Q) scans. The CT pulmonary angiogram (CTPA) demonstrated the existence and the extension of a filling defect in the pulmonary artery system. In the cases where V/Q scintigraphic scanning was employed, the results were interpreted by a nuclear medicine specialist. A high-probability V/Q scan was considered sufficient for the diagnosis of acute PE. This examination showed the presence of at least two segmental perfusion defects without ventilatory or radiological abnormalities in the same territories. ^2^^,^^15^ PE diagnosis was also assessed by a positive venous doppler ultrasound consistent with deep venous thrombosis (DVT) in patients with high clinical suspicion of PE and positive D-dimer values. ^2^ All CT, scintigraphy and venous doppler ultrasound scans were analyzed and interpreted by experienced specialists.

### Definitions

Complete RBBB was identified according to the Minnesota Code criteria (7-2-1) as a QRS duration must be ≥120 ms in addition to R′ wave>R wave in lead V1 and/or V2 in most beats of leads I, II, III, aVL, aVF; or a QRS complex being predominantly upright with an R-peak duration ≥60 ms in lead V1 and/or V2 or; an S wave duration > to R wave duration in all beats in lead I and/or II. ^16^^,^^17^

Incomplete RBBB was identified according to the Minnesota Code criteria (7-1) by a QRS duration in each of leads I, II, III, aVL, and aVF being <120 ms in addition to an R′>R wave in lead V1 and/or V2. ^16^^,^^17^

SIQIII-type ECG patterns were defined as a qualitative presence of S wave in lead I and Q wave in lead III.

Right ventricular dysfunction (RVD) was defined as a decreased systolic function of the RV (TAPSE <17 mm) and/or the presence of a paradoxical interventricular septal movement and/or a systolic pulmonary artery pressure ≥40 mmHg.

Major Adverse Cardiovascular Events (MACE) were defined as the presence of at least one of the following: death during hospitalization, cardiogenic shock in initial presentation or the presence of a thrombus in the right heart.

Renal failure was defined as an eGFR < 60 ml/min per 1.73 m ^2^. ^18^

Positive troponin levels were defined as any value above the 99th percentile of the upper reference limit. ^19^

Patients with PE with a systolic blood pressure (SBP) <90 mmHg at admission were classified as high-risk of mortality patients. ^2^

The sex of each participant was defined based on self-report and assigned following external examination of body.

### Inclusion criteria

Patients were eligible if: i) The diagnosis of PE was confirmed using one of the three diagnostic tools described above; ii) they were managed in Cardiology A Department, Fattouma Bourguiba University Hospital affiliated to the University of Monastir and the Cardiology Department of Kairouan affiliated to the University of Sousse; and iii) aged 18 years or above.

Exclusion criteria included: i) patients aged under 18 years and ii) if the diagnosis of acute PE was unclear.

A standard 12-lead ECG was assessed in the Emergency Department immediately upon initial contact. This first ECG taken at admission was selected for analysis. Both the ECG and echocardiography exams were performed and interpreted by experienced examiners. The retrospective analysis of ECG parameters focused on the existence of RBBB, either complete or incomplete, and SIQIII-type patterns.

### Statistics

Data analysis was performed using IBM SPSS Statistics (RRID:SCR_016479) version 26.0. The mean ± standard deviation (SD) was used to describe the normally distributed data. The median and interquartile range (IQR) was used to describe the skewed distribution data.

Frequencies and percentages were used to present categorical variables. The comparison between frequencies was performed using the Chi-squared test (χ ^2^ test) or Fisher’s exact test. Means were compared using the Student’s t test for independent samples. A Kaplan–Meier survival analysis was performed to assess the association between the RBBB and/or SIQIII and overall mortality using the log-rank test. In order to identify the independent factors associated with the occurrence of MACEs, univariate and multivariate analyses were performed. First, covariates with a p-value less than or equal to 0.20 were retained in the multivariable model. Then, a binary logistic regression was performed for assessing the independent risk factors for MACEs.

Associations were reported as adjusted Odds Ratios (aOR) with 95% Confidence Interval (95% CI). A p-value≤0.05 was considered as statistically significant.

## Results

A total of 255 patients were included in the analysis. All of the medical reports for these patients included a usable ECG from the time of their acute PE episode. Patients were divided into two groups: i) Group I (n=122, 47.8%) included patients with PE without RBBB nor SIQIII patterns; and ii) group II (n=133, 52.2%) included patients with RBBB and/or SIQIII type patterns. Patients in both groups did not differ by age nor sex. The proportion of diabetes mellitus (DM), hypertension (HTN) and dyslipidemia were homogeneous between the two groups ( Table 1). ^25^

**Table 1.** Patients characteristics. HTN, hypertension; VTE, venous thromboembolism; SBP, systolic blood pressure; DBP, diastolic blood pressure; TTE, transthoracic echocardiogram; IVS, intact ventricular septum; SPAP, systolic pulmonary artery pressure; RVD, right ventricular dysfunction; TAPSE, tricuspid annular plane systolic excursion; ECG, electrocardiography; RBBB, right bundle branch block; MACE, major cardiovascular event.

Characteristics	All	Group I (n=122)	Group II (n=133)	p-value
**General**				
Age, years, mean ± SD	61 ± 17.9	61.7 ± 18.5	60.3 ± 19.3	0.5
Male sex, n (%)	112 (43.9)	50 (41)	62 (46.6)	0.37
Diabetes mellitus, n (%)	53 (20.8)	25 (20.5)	28 (21.1)	1
HTN, n (%)	79 (31)	39 (32)	40 (30.1)	0.7
Dyslipidemia, n (%)	30 (11.8)	16 (13.1)	14 (10.5)	0.5
Smoker, n (%)	46 (18)	22 (18)	24 (18)	1
Active cancer, n (%)	38 (14.9)	15 (12.3)	23 (17,3)	0.29
History of VTE, n (%)	41 (16.1)	23 (18.9)	18 (13.5)	0.3
Heart failure, n (%)	24 (9.4)	6 (4.9)	18 (13.5)	0.03
**Clinical**				
Dyspnea, n (%)	203 (79.6)	89 (73)	114 (85.7)	0.13
Chest pain, n(%)	145 (56.9)	68 (55.7)	77 (57.9)	0.8
Cough, n (%)	25 (9.8)	8 (6.6)	17 (12.8)	0.13
Syncope, n (%)	30 (11.8)	11 (9)	19 (14.3)	0.24
Hemoptysis, n (%)	26 (10.2)	10 (8.2)	16 (12)	0.4
Right heart failure symptoms, n (%)	82 (32.1)	22 (18)	60 (45.1)	0.001
Cardiogenic Shock at admission, n (%)	47 (18.4)	5 (4.1)	42 (31.6)	<0.001
SBP, mmHg, mean ± SD	116.3 ± 17.9	122.8 ± 21.7	110.3 ± 23	<0.001
DBP, mmHg, mean ± SD	70.5 ± 13.3	73.9 ± 11.7	67.5 ± 13.9	<0.001
**Biology**				
Positive Troponin n (%)	97 (38)	29 (26.9)	68 (51.1)	<0.001
Renal failure n (%)	81 (31.8)	33 (27.3)	48 (36.1)	0.13
**TTE parameters**				
SPAP ≥ 40 mmHg, n (%)	111 (43.5)	32 (28.3)	79 (63.2)	<0.001
Paradoxical IVS, n (%)	94 (36.9)	30 (26.5)	64 (51.6)	<0.001
Right heart thrombus, n (%)	39 (15.3)	8 (7.1)	31 (25)	<0.001
RVD, n (%)	136 (53.3)	43 (38.7)	93 (69.9)	<0.001
TAPSE <17 mm, n (%)	54 (29)	4 (4.2)	50 (55.6)	<0.001
**ECG**				
RBBB, n (%)	101 (39.6)	0	101 (75.9)	
SIQIII, n (%)	66 (26)	0	66 (49.6)	
RBBB and SIQIII, n (%)	34 (13.4)	0	34 (25.6)	
Atrial fibrillation, n (%)	23 (9)	13 (10.6)	10 (7.6)	0.4
Negative T wave, n (%)	117 (55.7)	42 (45.7)	75 (63.6)	0.012
**Other**				
MACE, n (%)	71 (27.8)	16 (13.7)	55 (43.3)	<0.001
In-hospital deaths, n (%)	40 (15.7)	10 (8.2)	30 (22.6)	0.002
Thrombolysis, n (%)	108 (42.7)	46 (37.7)	62 (47.3)	0.12

Group II patients had an active cancer (17.3% *vs.* 12.3%) more frequently but this difference did not reach statistical significance (p=0.29). Patients presenting with SIQIII pattern and/or RBBB were also significantly more likely to have history of systolic or diastolic heart failure (13.5% *vs.* 4.9%, p=0.03).

We analyzed the association between the presence of RBBB and/or SIQIII type pattern with PE severity and patient outcome.

Patients in group II presented more frequently with acute right heart symptoms (45.1 *vs.* 18%, p<0.001), more often showed cardiogenic shock (31.6 *vs.* 4.1%, p<0.001) and had lower systolic blood pressure (SBP) (110.3 *vs.* 122.8 mmHg, p<0.001) and diastolic blood pressure (DBP) (67.5 *vs.* 73.9 mmHg, p<0.001) at admission. The difference was statistically significant for all these parameters. Syncope occurred more frequently in Group II patients but the difference did not reach statistical significance (14.3 *vs.* 9%, p=0.24) ( Table 1).

Patients in group I had a significantly lower likelihood of positive Cardiac troponin I (cTnI) or high-sensitivity cardiac troponin (hs-cTn) levels (26.9 *vs.* 51.1%, p<0.001).

Echocardiographic parameters indicating a right heart injury also occurred more frequently in group II patients: SPAP >40 mmHg (63.2 *vs.* 28.3%, p<0.001), paradoxical interventricular septum (51.6 *vs.* 26.5%, p<0.001), tricuspid annular plane systolic excursion (TAPSE) <17 mm (55.6 *vs.* 4.2%, p<0.001), the presence of a thrombus in the right heart (25 *vs.* 4.2%, p<0.001). Thus, RVD was significantly more frequent in Group II patients by univariate analysis (p<0.001).

A total of 101 patients (39.6%) had RBBB, 66 (26%) had SIQIII type patterns, and 34 patients (13.4%) had both SIQIII pattern and RBBB.

Negative T waves in leads V1-V3 were significantly more frequently associated with Group II patients in univariate analysis (p=0.01) ( Table 1).

There were slightly more patients with atrial fibrillation (AF) in Group I patients, but this was not statistically significance (10.6 *vs.* 7.6%, p=0.4).

Groupe II patients were significantly associated with in-hospital mortality (22.6 *vs.* 6.1%, p=0.002) and MACEs during hospitalization by univariate analysis (43.3 *vs.* 13.7%, p<0.001) ( Table 1).

Multivariate logistic regression analysis demonstrated five independent predictors of MACE: SIQIII and/or RBBB (OR=2.4; 95% CI: 1.04, 5.9; p=0.039), renal failure (OR=2.5; 95% CI: 1.08, 5.8; p=0.031), positive troponin levels (OR=5.4; 95% CI: 2.34, 12.6; p<0.001), RVD (OR=7.9; 95% CI: 2.16, 28.8; p=0.002) and right heart failure symptoms during initial presentation (OR=2.5; 95% CI: 1.1, 5.8; p=0.025) ( Table 2). Using these models, SIQIII pattern (OR=4.8; 95% CI: 1.9, 12.2; p=0.001), RVD (OR=15.2; 95% CI: 1.9, 12.4; p=0.01) and cardiogenic shock at admission (OR=2.7; 95% CI: 1, 6.9; p=0.034) were independently associated with in-hospital mortality, on the other hand, no association was found between in-hospital mortality and group II patients ( Table 3).

**Table 2.** Independent predictors of MACEs. MACE, major cardiovascular event; RBBB, right bundle branch block; RVD, right ventricular dysfunction; OR, odds ratio; CI, confidence interval.

Variables	Multivariate		
	OR	95% CI	p-value
SIQIII and/or RBBB	2.4	1.04, 5.9	0.039
Renal failure	2.5	1.08, 5.8	0.031
Positive troponin	5.4	2.34, 12.6	<0.001
RVD	7.9	2.16, 28.8	0.002
Right heart failure	2.5	1.1, 5.8	0.025

**Table 3.** Independent predictors of in-hospital mortality. RVD, right ventricular dysfunction; OR, odds ratio; CI, confidence interval.

Variables	Multivariate		
	OR	95% CI	p-value
SIQIII	4.8	1.9, 12.2	0.001
Renal failure	3.8	1.5, 9.6	0.003
RVD	15.2	1.9, 12.4	0.01
Cardiogenic shock	2.7	1, 6.9	0.034

**Figure 1.**
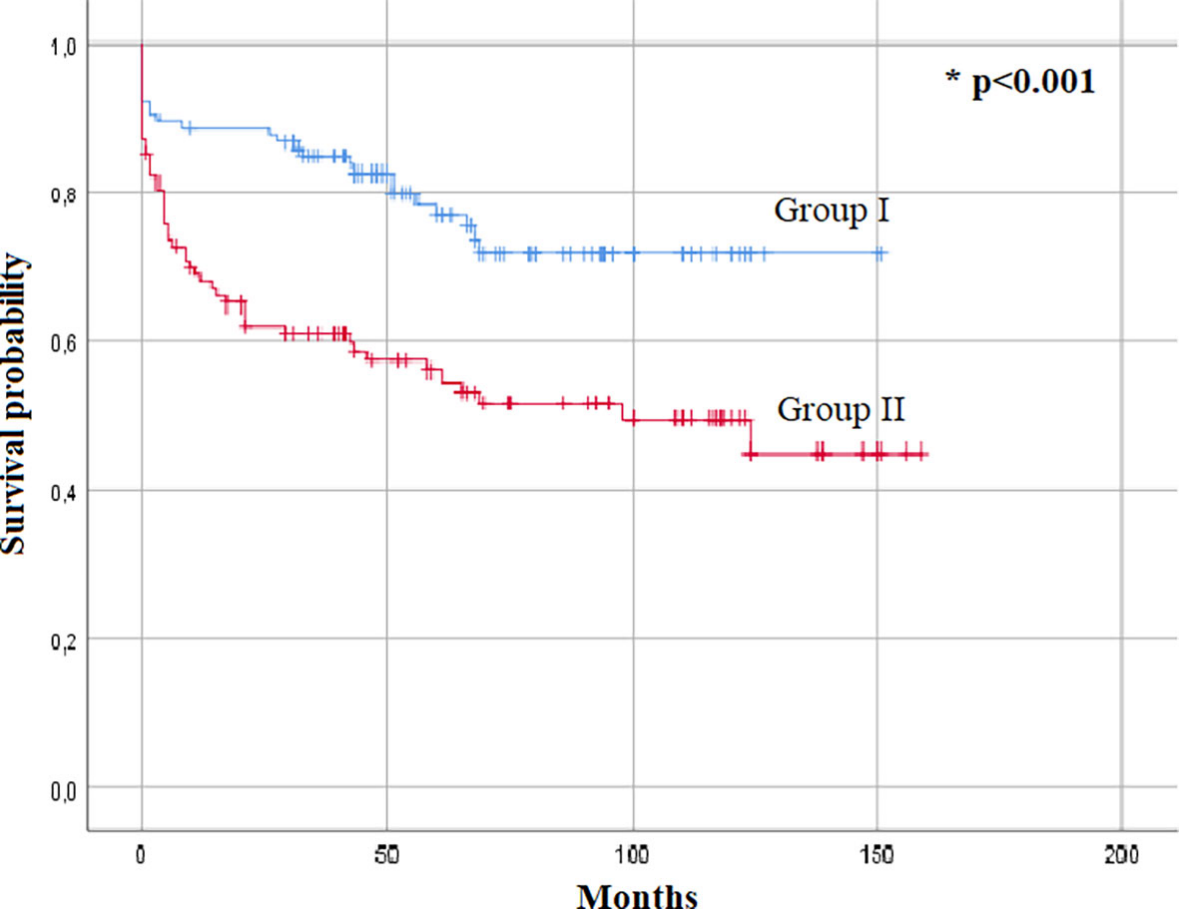
Kaplan–Meier survival estimates for both groups.

**Figure 2.**
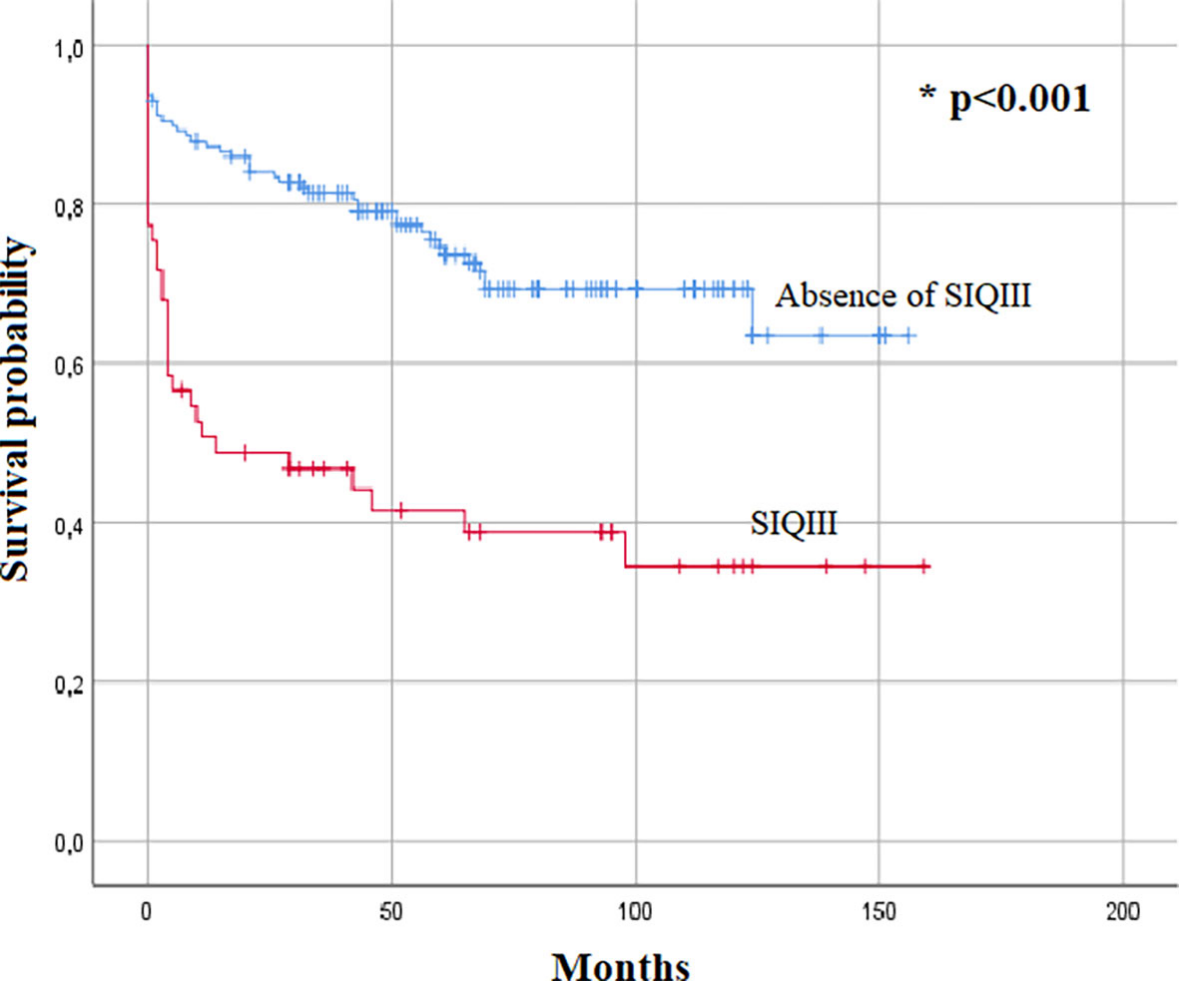
Kaplan–Meier survival estimates for SIQIII pattern patients.

A medium or long term follow-up was done for 83% of the cohort. The Kaplan–Meier survival analysis also demonstrated SIQIII patterns as a predictor of overall mortality (p<0.001) ( Figure 1). The Kaplan–Meier analysis also identified the presence of SIQIII and/or RBBB patterns as a predictive factor for overall mortality (p<0.001) ( Figure 2).

## Discussion

Early and risk-oriented diagnosis and management of patients with acute PE are key for a better prognosis. ^2^ In our study, RBBB and SIQIII-type patterns at admission were found to be associated with worsening clinical, echographic and biological profiles and with a poor clinical outcome.

Meta-analyses by Shopp *et al.*, reported different ECG patterns, such as inverted T wave in leads V1-V4, a QR pattern in lead V1, SIQIII, complete or incomplete RBBB and ST elevation in lead aVR as being associated with increasing severity and poor outcomes. ^4^

ECG scoring by Daniel *et al.*, was notable for providing an applicable and reliable prognostic tool. ^5^ However, recent studies indicate that there are several ECG abnormalities that can provide valuable prognostic information, but are not currently included in these scores. ^9^ These ECG findings were also associated with early clinical deterioration. ^20^ Despite these findings, ECG abnormalities were not included nor recommended in the latest guidelines for risk stratification of acute PE. ^2^ This is probably due to the lack of specificity as these changes can be encountered in acute and chronic cor pulmonale. ^21^

The SIQIII pattern was found to be a strong independent predictor of in-hospital mortality, contrary to RBBB. Acute onset of SIQIII patterns mirror a longitudinal dextrorotation of the RV and appears to be more associated with poor hemodynamic outcomes than RBBB. RBBB alone was not found to be independently associated with in-hospital mortality The findings concerning RBBB were interpreted as follows: this characteristic can be observed in both acute pulmonary embolism (PE) and different conditions affecting the right ventricle (RV). Consequently, distinguishing between a new onset RBBB and a preexisting chronic RBBB is challenging. Furthermore, this challenge may be attributed to the limited sample size in various studies exploring the correlation between ECG abnormalities and risk assessment in acute PE.

New onset SIQIII and/or RBBB is likely to increase right heart failure, cardiogenic shock and intra-hospital mortality. These ECG changes seem to profoundly impact the overall survival as patients with SIQIII and/or RBBB patterns, and especially those with SIQIII, have significantly lower survival rates with curves diverging in less than a year. Parallel to the Pulmonary Embolism Severity Index (PESI) and to the simplified Pulmonary Embolism Severity Index (SPESI) scores, these findings suggest the importance of the RBBB and SIQIII patterns as important criteria in risk stratification of PE. ^12^ The recent onset of these patterns may improve its specificity especially if other causes of acute cor pulmonale are ruled out.

Given the predictive value of these parameters, it would be beneficial to integrate them into new and more accurate risk stratification scores for future guidelines. This would probably outperform guideline-backed risk scores. ^22^

Furthermore, our study suggests that ECG alone is a useful tool in determining the outcome of PE, particularly in limited resources environments where advanced diagnostic tools are not available. ^23^^,^^24^

## Conclusions

Risk stratification is key for the management of patients with acute PE. New-onset RBBB and/or SIQIII and especially SIQIII patterns are likely to worsen patient outcomes. Our study suggests important and independent prognostic values of RBBB and SIQIII patterns and their usefulness in determining the outcome of acute PE patients.

## Data Availability

Figshare: EP registre 2023 MONASTIR _ KAIROUAN final - SAFE HARBOR.sav. https://doi.org/10.6084/m9.figshare.22153379. ^25^ Data are available under the terms of the Creative Commons Attribution 4.0 International license (CC-BY 4.0).
