# Supplementary material for: The impact of right bundle branch block and SIQIII-type patterns in determining risk levels in acute pulmonary embolism
Source: F1000Res. 2023 May 24;12:545. [Version 1] doi: 10.12688/f1000research.131758.1 (PMC11134142; doi:10.12688/f1000research.131758.1)
Supplement: Peer review history

# Reviewer report: Reviewer response for version 1

**DOI:** 10.5256/f1000research.144628.r279907

**Published:** 2024-05-28

**Authors:** Boris Dzudovic

**Affiliations:** 1 Clinic for Emergency Internal Medicine, Military Medical Academy, University of Defense, Belgrade, Serbia

Summary:

 The study investigates the prognostic significance of right bundle branch block (RBBB) and SIQIII patterns on the risk stratification and outcomes of patients with acute pulmonary embolism (PE). Conducted as a retrospective cohort study involving 255 patients over a period of 11 years, the research provides valuable insights into the association between these ECG patterns and clinical outcomes, emphasizing their potential utility in risk stratification.

 Review:

 1. **Is the work clearly and accurately presented and does it cite the current literature?**

**Yes.** The manuscript is well-written and clearly presents its findings. It adequately references current and relevant literature, contextualizing its contributions within the existing body of knowledge.

 2. **Is the study design appropriate and is the work technically sound?**

**Yes.** The study design is appropriate for the research question. The use of a bicentric study design with a substantial patient cohort enhances the robustness of the findings. The methods, including statistical analyses, are solid and align with the study objectives.

 3. **Are sufficient details of methods and analysis provided to allow replication by others?** **Yes.** The methods section is detailed, allowing for reproducibility. The descriptions of ECG criteria, inclusion/exclusion criteria, and statistical methods are thorough and clear.

 4. **If applicable, is the statistical analysis and its interpretation appropriate?**

**Yes.** The statistical analyses are appropriate, with a comprehensive use of univariate and multivariate logistic regression analyses to identify independent predictors of major adverse cardiovascular events (MACEs) and in-hospital mortality. Kaplan-Meier survival analyses further enrich the interpretation of the data.

 5. **Are all the source data underlying the results available to ensure full reproducibility?**

**Yes.** The underlying data is available as cited, ensuring transparency and reproducibility.

 6. **Are the conclusions drawn adequately supported by the results?**

**Yes.** The conclusions are well-supported by the data presented. The study effectively demonstrates the prognostic value of RBBB and SIQIII patterns in patients with acute PE.

 Specific Comments:

**Definition of MACE:**

 - The inclusion of right heart thrombus as part of the MACE definition is debatable as it may not always lead to major cardiovascular events and can sometimes be asymptomatic. A clearer rationale for this inclusion or a standardized definition would enhance the robustness of the findings.

**SPAP Calculation:**

 - The calculation of systolic pulmonary artery pressure (SPAP) when tricuspid annular plane systolic excursion (TAPSE) is <17 mm requires clarification. The potential underestimation of PAP using tricuspid regurgitation flow in these cases should be addressed, as it impacts the interpretation of right ventricular dysfunction.

**Ethical Considerations:**

 - While the study is retrospective and received ethical approval, the initial lack of ethical approval and subsequent retrospective approval should be more transparently discussed, especially regarding the collection of verbal consent from critically ill patients.

 Minor remarks:

 1. The study provides valuable contributions to the field and has the potential to influence clinical practice by incorporating ECG findings into risk stratification models for PE.

 2. The presentation of survival curves adds a significant dimension to understanding the prognostic implications of the ECG patterns studied.

**Conclusion**:

 The article is a significant contribution to the literature on PE and its risk stratification. The findings support the integration of RBBB and SIQIII patterns into risk stratification algorithms, potentially improving patient management. Addressing the minor issues noted would further strengthen the study.

**Recommendation**:

**Accept with minor revisions.**

Is the work clearly and accurately presented and does it cite the current literature?

Yes

If applicable, is the statistical analysis and its interpretation appropriate?

Yes

Are all the source data underlying the results available to ensure full reproducibility?

Yes

Is the study design appropriate and is the work technically sound?

Yes

Are the conclusions drawn adequately supported by the results?

Yes

Are sufficient details of methods and analysis provided to allow replication by others?

Yes

Reviewer Expertise:

acute cardiac conditions includin acute PE and CTEPH.

I confirm that I have read this submission and believe that I have an appropriate level of expertise to confirm that it is of an acceptable scientific standard.

# Reviewer report: Reviewer response for version 1

**DOI:** 10.5256/f1000research.144628.r210932

**Published:** 2023-10-16

**Authors:** Talel Trimech

**Affiliations:** 1 Rambouillet Hospital Center, Le Chesnay, France

I found the article to be interesting. The exploration of ECG modifications and their correlation with acute pulmonary embolism risk stratification has been a matter of several debates. Using such simple methods, if proven accurate, might sharpen the sensibility of such predictive models.

 The bicentric study design with a substantial number of patients adds considerable weight to the findings, enhancing the credibility of the study and positioning it as one of the most credible papers addressing the relationship between ECG modifications and risk stratification. The statistical analysis carried out in the study has solid standards, and the inclusion of survival curves added a valuable dimension to the interpretation of the data.

 I therefore, have two minor remarks:

- The definition of MACE included the presence of a thrombus in the right heart. This can be asymptomatic and not leading to a major cardiovascular event. Although the definition of MACE has not been standardized, I think that the inclusion of such TTE findings might be a matter of discussion.
- How were the SPAP calculated when the TAPSE was <17 mm. Using the tricuspid regurgitation flow might underestimate the PAP values in such cases.

Overall, I commend the authors for their work in conducting this study and presenting the results in a clear and impactful manner. This research has the potential to influence clinical practice positively.

 Thank you for considering my feedback.

 Best regards

Is the work clearly and accurately presented and does it cite the current literature?

Yes

If applicable, is the statistical analysis and its interpretation appropriate?

Yes

Are all the source data underlying the results available to ensure full reproducibility?

Yes

Is the study design appropriate and is the work technically sound?

Yes

Are the conclusions drawn adequately supported by the results?

Yes

Are sufficient details of methods and analysis provided to allow replication by others?

Yes

Reviewer Expertise:

Cardiovascular diseases, heart failure, pulmonary embolism

I confirm that I have read this submission and believe that I have an appropriate level of expertise to confirm that it is of an acceptable scientific standard.
